# "Get off the sofa and go and play": Family and socioeconomic influences on the physical activity of 10–11 year old children

**DOI:** 10.1186/1471-2458-9-253

**Published:** 2009-07-21

**Authors:** Rowan Brockman, Russell Jago, Kenneth R Fox, Janice L Thompson, Kim Cartwright, Angie S Page

**Affiliations:** 1 Department of Exercise, Nutrition and Health Sciences, University of Bristol, Bristol, UK; 2 School of Psychology, University of Southampton, Southampton, UK

## Abstract

**Background:**

Physical activity declines as children approach puberty. Research has focussed on psychosocial, environmental, and demographic determinants. This paper explores how family and socioeconomic factors are related to children's physical activity.

**Methods:**

Seventeen focus groups were conducted with 113, 10–11 year old children from 11 primary schools in Bristol, UK. Focus groups examined: 1) the way parents encourage their children to be physically active; 2) the extent to which physical activity is engaged in as a family; and 3) the types of non-family based physical activities Year 6 children commonly participate in.

**Results:**

Participants from all socioeconomic (SES) groups reported that parents encouraged them to be physically active. However approaches differed. Children from middle/high SES schools were assisted through actions such as logistical and financial support, co-participation and modelling. Parents of children from low SES schools mainly restricted their input to verbal encouragement and demands. Participation in family-based activities was reported to be higher in children from middle/high SES schools than children from low SES schools. All SES groups reported time to be a limiting factor in family-based activity participation. Cost was reported as a significant barrier by children from low SES schools. Children from middle/high SES schools reported engaging in more sports clubs and organised activities than children from low SES schools, who reported participating in more unstructured activities or 'free play' with friends.

**Conclusion:**

The family is important for encouraging children to be physically active, but families from different socioeconomic backgrounds support their children in different ways. This research suggests that the design of physical activity interventions, which might include working with families, requires tailoring to groups from different socio-economic backgrounds.

## Background

Physical activity during childhood and adolescence provides many short and long-term health benefits. In addition to reducing risks for high blood pressure [1] and dyslipidaemia [2], higher levels of physical activity in children are associated with a decreased risk of adiposity [3]. A number of studies have shown that many youth do not meet physical activity guidelines [4]. Moreover, physical activity declines and sedentary behaviour becomes more common between the ages of 10–12 [5]. Taken together, these findings highlight the need to identify the factors that contribute to children's participation in regular physical activity at this age.

Many factors have been associated with youth physical activity including psychosocial factors such as self-efficacy and activity preference [6] and aspects of the physical environment [7]. However, little research has focused on family influences on physical activity. Emerging evidence suggests that the family is an important provider of social support for children and adolescents' physical activity [8-10], however, some research has questioned the strength of this relationship [11,12]. Such inconsistencies in the literature provide a strong rationale for more exploratory qualitative research.

There is conflicting research on *how *families influence children's physical activity patterns. For example, one study discussed the plausibility of parents acting as role models for children's physical activity [13] whilst a recent review of the correlates of physical activity found parental encouragement and support to be more strongly associated with children and adolescents' participation in physical activity than parental role modelling [14]. Further research is necessary to assist in the development of strategies to change family behaviours.

Adult physical activity has been shown to differ by socioeconomic status (SES) [15], but it is not known at what age this difference emerges. Data from the Scottish Health Behaviour Survey [16] showed that, in a sample of 11–15 year olds, as family affluence increased, so did levels of self-reported vigorous physical activity (VPA), with girls taking part in less activity than boys across all socioeconomic groups. A longitudinal study in a UK sample of secondary school children showed marked reductions in physical activity and increases in sedentary behaviour between Years 7–11(ages 11–16) and that levels of sedentary behaviour were greater in respondents from lower SES households [5].

In addition to research showing SES differences in the frequency of physical activity, some research has shown differences in *types *of activity between SES groups. For example, Finnish data [17] has shown that high family income is associated with the increased likelihood of adolescents being active sports club members. These findings are reinforced by US data [18], which showed that high SES adolescents were more likely to be involved in organized physical activity programmes than lower income groups.

There is a shortage of comparable information for UK children and there is a need to understand how SES is associated with the types of activities in which children engage. Therefore, the aim of this study was to explore the influence of family and socioeconomic factors on children's physical activity. This aim was addressed by analysing qualitative data based on focus groups with children from schools in different economic areas of Bristol, UK.

## Methods

A total of 113, 10–11 year old children were recruited from 11 primary schools in Bristol, UK. The schools represented the socio-economic diversity of the local area based on the Index of Multiple Deprivation (IMD). The IMD is a UK Government-produced measure of deprivation that includes assessments of income, employment, health and education [19]. The IMD was obtained for the postcode of each school and thus represented a measure of deprivation for the school and not the individual participant. Based on the IMDs for the postcodes of all schools within a 10 mile radius of the University of Bristol, four schools were recruited from the lowest third (Low SES schools), four from the middle third (Middle SES schools) and three from the highest third (High SES schools). A 'recruitment' session was held for all Year 6 pupils (10–11 years of age) in each school, in which children were invited to participate in a research study about physical activity and their friends and family. The study was approved by the School of Applied Community and Health Studies Ethics committee at the University of Bristol (ref 017/06) and informed parental consent and childhood assent were obtained for all participants [20].

Focus groups were chosen as the method of data collection. Focus groups are an effective method of collecting qualitative data from children as the thoughts and ideas of other members of the group often help participants verbalise their responses in a comfortable, safe and supportive environment [21,22]. Depending on the number of consenting participants, one or two focus groups were held at each school, with a range of 2–12 children in each group. Each focus group lasted 30–45 minutes, and was conducted by a trained moderator and was recorded using an Olympus DS-2200 Digital recorder. The focus groups had a semi-structured design with follow-up probes on key topics of interest. Questions were developed by the study team and piloted in another school before being finalised for the current research. Questions focused on the kinds of strategies parents use to help their children be more physically active (e.g. "Is there something that a parent/carer or other family member could do to help you be more physically active?" – prompts included providing transport to after-school clubs and encouraging outside activity); the extent to which physical activity is engaged in as a family (e.g. "If you do physical activity with your parents/carers and other family members, what types do you do?") and types of activities in which the children engaged and with whom (e.g. "If you take part in physical activity after school, what types do you do and where do you do it?").

### Analyses

All recordings were transcribed verbatim and anonymised. A second researcher listened to the recordings and checked the transcripts for accuracy with a third researcher reconciling any differences. Analyses were conducted in two phases. First, key themes were identified by reading the transcripts line by line and marking the text with codes that described the content of the response [23]. Codes were entered as 'free nodes' (labels that describe themes) into a newly created database in NVivo (Version 2.01, QSR, Southport UK). Codes were checked by a second investigator and then put into a hierarchical format. Second, to assess whether there were SES differences in responses, text retrievals on each key code were performed separately by SES group and summarized to provide an overview of the themes within each group.

## Results

Participant characteristics are shown in Table 1. Seventeen focus groups were conducted and the sample was 52% female. Participant responses were based around three themes: 1) whether and how parents encourage children to be physically active; 2) the extent to which physical activity is engaged in as a family and barriers to family participation 3) the prevalence of active play versus organised sports in Year 6 children's leisure time physical activities. Initial analysis of the themes by SES of school indicated that that children from middle and high SES schools expressed very similar views and therefore these two groups were merged into a 'middle/high SES' group. A summary of the three themes is presented in Table 2 and discussed below.

**Table 1:** Participant characteristics

**School SES**	**No. of Schools**	**No. of Focus Groups**	**N**	**Boys n (%)**	**Girls n (%)**
Low	4	5	27	15 (55.5)	12 (44.5)
Middle	4	6	41	17 (41.5)	24 (58.5)
High	3	6	45	22 (48.9)	23 (51.1)
**Total**	**11**	**17**	**113**	**54 (47.8)**	**59 (52.2)**

**Table 2:** Summary of key themes identified from focus groups and how they differ by SES group

**Theme**	**Key items for Low SES Schools**	**Key items for Middle/High SES Schools**	**Key items for all SES Schools**
**1) Parental encouragement of physical activity**	Parents encourage their children to take part in physical activity They do this through verbal demands	Middle/high SES parents encourage their children to take part in physical activity They do this through non-verbal, proactive methods, including logistical support, financial support and co-participation	Parents encourage their children to take part in physical activity
**2) Activity as a family: Barriers to participation**	Less participation than middle/high SES groups in family-based activity Money is viewed as a key factor affecting participation in physical activity	More participation in family-based activity than low SES groups	Time is perceived as a factor influencing participation in family-based activity
**3) Types of activity in which children engage: Active play versus organised sports**	Children engage in more unstructured physical activity/'free play' than those from middle/high SES schools	Children participate in more organized/structured activity, such as after-school and weekend sports clubs	

### Parental encouragement of physical activity

Most participants reported that their parents encouraged them to engage in physical activity but the way in which encouragement was manifested appeared to differ according to the SES of the school. Children from middle/high SES schools mainly reported that they were encouraged by their families to take part in physical activity through non-verbal methods which included logistical and financial support, modelling and co-participation. For example:

"... my parents do all they can to persuade me...to do physical activities and they take me to or from if, needed, and hopefully they'll do the same to my sisters when they start..." (Female, middle/high SES)

"Mum and dad help me by buying me sports equipment." (Female, middle/high SES)

"... my mum like encourages me to do sport because she's quite sporty." (Female, middle/high SES)

"... my mum and dad encourage me, my dad does incredible a lot of walking, so I try to keep up." (Male, middle/high SES)

In contrast, children from lower SES schools mainly reported that they are encouraged by their families to take part in physical activity through *verbal demand *methods:

"My mum says to get off the sofa and go and play tennis or something." (Female, low SES)

"... they tell me to get off the x-box and say like if you don't do the activity you're not going on screens..." (Male, low SES)

"My dad's always telling me to go out and do something." (Female, low SES)

"Sometimes I can't be bothered to go to rugby so my dad forces me, not literally forces me, but says I have to go." (Male, low SES)

### Activity as a family: Barriers to participation

Although children from all SES schools felt that their parents encouraged them to be physically active, children from middle/high SES schools reported participating in physical activity more often with their families than children from low SES schools:

"Well my mum or my dad takes me swimming and we all go dog-walking and occasionally we go and play squash at [name of local sport centre] and go for cycle rides." (Female, middle/high SES)

"Well in the summer we like...my mum likes us to do lots of things that are family, I think it's like 'family bonding time', I don't know, and um we go sailing, except for my mum stays at home cos she's scared and ...oh and my dad sometimes takes me swimming. (Female, middle/high SES)"

"I do ice-skating with my mum and my cousin sometimes, but that's like once every two months." (Female, low SES)

"Well I, I do do some with my cousin...I always, well, basically I don't do that much with my family." (Male, low SES)

Participants reported that they are limited in taking part in family-based physical activity by their parents' lack of free time. This limitation affects children of all SES schools:

"...my dad usually goes to work quite like, early and he comes back quite late...he usually just takes me swimming just like once a week...and my mum doesn't have much time to take me..." (Female, middle/high SES)

"... I do physical activities with my mum in the holidays but my step dad works when my mum looks after me and we and me and my brother, well brothers, I got three brothers, um we go cycling sometimes and my step dad some does when he gets time off." (Female, low SES)

Another factor which affects family-based physical activity is cost, but this was only reported by children from low SES schools.

"...we can't really do much because I can't help it that they haven't got a lot of money..." (Female, low SES)

"There are certain people that do quite a lot of activities, well depending on how much money they've got." (Female, low SES)

### Types of physical activity in which participants engage: Active play versus organised sports

Participants engaged in different types of physical activity after-school and at the weekend, according to SES of school. Physical activity for children from middle/high SES schools tended to be organised and based around after-school and/or weekend sports clubs:

"I do something every day, I do, on Monday I go to synchronised swimming, on Tuesday I go swimming with the school, and we do we did girls cricket, on Wednesday I go to tap, on Thursday I go to Jazz, on Friday I go to Synchro, Saturday I go swimming and Sunday I go to Synchro." (Female, middle/high SES)

"WelI I go to quite a lot [of after-school clubs] cos my mum and dad don't finish work until quite late so I try and get into doing... football and cricket and I do swimming sometimes." (Male, middle/high SES)

"Monday, me and [child's name] walk up to [name of local secondary school] and do drama and Tuesday I do netball here, Wednesday I do football here, Thursday I don't do anything, Friday I do tennis in [name of a neighbouring village] and I might be, I'm starting to play for [name of a neighbouring village]... football and tennis in [name of neighbouring village] on Fridays and Sunday I do rugby at [name of local rugby club]." (Male, middle/high SES)

In contrast, children from low SES schools engage in more unstructured physical activity, e.g. 'active play' in the park or in the streets with friends:

" [I] hang around with my friends on street corners cos you know when it was raining last week, I was out in that, with no coat on, I was jumping around in puddles." (Female, low SES)

"... usually on the weekends I just go out on my bike and ride around with my friends or...my cousins and family." (Male, low SES)

"We go out and play football or we ride around on our bikes, or if it's too cold we just play inside and do something." (Female, low SES)

"On Monday we went on our bikes, Tuesday we went to go and play football, Wednesday we went and played catch." (Male, low SES)

The implications of this are that children from low SES schools are spending more time with friends in an unstructured setting, which is potentially unsupervised by adults. In contrast, the physical activity of children from middle/high SES schools tends to be highly structured and rule-based, with a clear adult presence. Indeed, these children often viewed unstructured activities as a better opportunity to be with friends than organized clubs, which focused on 'playing sports':

"I do like playing with my friends, but I do have a lot of football that I have to do so that kind of messes things up sometimes." (Male, middle/high SES)

"If I'm not going to a club usually I like to cycle around with my friends and we go on trips around [name of local area] on our bikes." (Female, middle/high SES)

"I would probably prefer to see like friends and usually part of seeing friends is like going outside and playing football so it's half wanting to do it more than playing sports." (Male, middle/high SES)

"I would prefer like not to do it as a club just do it with my friends like instead of doing a swimming club just go swimming." (Female, middle/high SES)

## Discussion

This study explored the influence of family and socioeconomic factors on the physical activity of 10–11 year old children. The data presented in this paper indicate that the way in which parents encourage children to be physically active, the extent to which physical activity is engaged in together as a family and the types of non-family based physical activities in which children engage, differ by the SES of the schools which the children attend. Children from low SES schools are mainly verbally encouraged to take part in activity, in a demanding or directive way. It is not clear from this study that this type of encouragement was directly related to children's physical activity, as data regarding activity levels of the children was not collected. However, one explanation could be that low SES families rely more on verbal encouragement due to financial constraints on transport, sports equipment and enrolment in sports clubs, which families of middle/high SES may not face.

Children attending middle and high SES schools are non-verbally encouraged to take part in activity, mainly by parents' financial support, co-participation and modelling. This is consistent with previous research which identified maternal and paternal logistical support and maternal and paternal modelling as two of the seven sources of activity-related support for children from predominantly middle SES families [9]. This result is significant because it may help to clarify the most effective targets for health promotion and public health interventions.

The verbal strategies employed by parents of children from low SES schools to encourage the children to engage in physical activity, via rewards or sanctions, are consistent with extrinsic motivation. Given that intrinsic motivation has been associated with physical activity adoption and maintenance among children and adults [24,25] this approach may not be the most effective means of increasing children's participation in physical activity. Working with lower SES families to build intrinsic motivation for children's physical activity and facilitating a mindset whereby children "want to exercise" rather than "ought to exercise" could be an effective means of increasing youth physical activity.

Children from middle and high SES schools reported engaging in family-based physical activity more often than children from low SES schools. Time was a factor that appeared to constrain family-based activity regardless of SES group, but money only appeared to constrain low SES groups. Lack of time has previously been reported by youth as a significant barrier to physical activity [26] and cost has been reported as a barrier by adults [27]. Thus, there is a need to provide information on how families can more efficiently and economically build physical activity into their everyday lives. A good example would be promoting walking or cycling to school which, aside from requiring little or no extra time commitment, is lower in cost than motorised transport.

The data also suggest that SES has an influence on the types of physical activity in which children engage, with children from middle and high SES schools engaging in more structured activity, such as after-school and weekend sports clubs, and children from low SES schools engaging in more 'active play'. The findings provide support for previous research, which found that participation in organised sports clubs was greater in higher SES/income families [17,18].

Participation in youth sport and other organized physical activities can be a considerable financial cost to parents and may be problematic for families from low SES schools [18]. Engagement in structured activities can provide access to coaching and help build competence and a sense of being part of a team in young children if done well [28]. To enable equal opportunities to participate in such activities there is a need to find ways to subsidise participation for all youth. Additionally, there is a need to promote opportunities for increased participation in more unstructured activity, or 'active play', which is accessible to children from all SES backgrounds. This is because recent research suggests that active play may potentially be a major contributor to total physical activity levels [29].

One way this could be achieved is by improving children's access to safe play areas in their local community, such as parks, fields and other green space and also, improving existing play facilities within these areas. Indeed, findings from a recent study suggest expanding park access and safety, particularly for youth living in urban areas, is a promising strategy for increasing their physical activity [30]. Play England called for the introduction of planning requirements for outdoor play space in all new housing builds and re-designs; more effective traffic calming in residential areas and an increase in the number and variety of places for children to play including staffed and unsupervised dedicated play provision [31]. Once these initiatives have been implemented their effect on physical activity needs to be evaluated.

A number of studies have focused on differences in the amount of physical activity children engage in, but much less work has examined the factors that influence activity type. This study found that the type of activities in which youth engage differ by the SES of school, with children from middle/high SES schools engaging in more structured physical activity and children from low SES schools in more 'active free play'. Ideally, we want children from all SES groups to be given access to the full range of physical activity opportunities, as different settings provide different learning and developmental experiences. Organised leisure activities are sometimes seen as better for developing the creativity of the child than playing in the street without adult control [32], whereas unstructured play offers other benefits in terms of dealing with social conflict, managing risk and developing autonomy [33]. Indeed, recent UK Government guidance on how local authorities and their partners can promote physical activity asked that "children...experience a wide range of formal and informal activities both in and out of school, from walking to school, to...active free play in well-maintained open spaces" [34]. This suggests that the development of pilot interventions to increase activity for children in a variety of contexts and a range of socioeconomic backgrounds is warranted.

### Limitations

This study focused on the extent to which a few potentially important factors influenced youth physical activity. However, as participants were not asked about other school- and community-level environmental influences we cannot assess how the family level factors were associated with these variables. It is also important to recognize that the SES data was based on the IMD scores of the school postcode which, because children can sometimes live in a different geographical ward to their school, may not accurately reflect the SES of individual participants. Additionally, the IMD is an aggregate score based on various characteristics of the neighbourhood, and not solely on family income or education level. Also, it must be noted that the data reported here relied on children's perceptions of their own and their families' physical activity, which may not be an accurate representation of reality. Finally, the role of other family members in children's physical activity was not a specific focus of our questioning, and, as a result, they were rarely mentioned. We recognise that some family members, such as grandparents and siblings, may play an important role in influencing children's physical activity and acknowledge that further research into their influence on Year 6 children's physical activity may be required.

## Conclusion

Findings from this study suggest that families think about, communicate and play an important role in promoting the physical activity of their children. The study also suggests that the way in which families encourage their children to be active differs according to socioeconomic background, with children from middle and high SES schools reporting more proactive methods by parents, and children from low SES schools restricted to verbal exchange. Finally, interventions involving the promotion of time efficient, low-cost activities such as active travel to work and school, may help to increase family physical activity across the socioeconomic spectrum and unstructured, 'free play' may be an important way of increasing physical activity participation in children from lower socioeconomic backgrounds.

## Competing interests

The authors declare that they have no competing interests.

## Authors' contributions

The study was designed by RJ, KF, AP and JT. Analysis was performed by RB and KC. The first draft of the paper was written by RB and all authors provided critical input and revisions.

## Pre-publication history

The pre-publication history for this paper can be accessed here:


